# Consumption of Sugar-Sweetened Beverages Has a Dose-Dependent Effect on the Risk of Non-Alcoholic Fatty Liver Disease: An Updated Systematic Review and Dose-Response Meta-Analysis

**DOI:** 10.3390/ijerph16122192

**Published:** 2019-06-21

**Authors:** Hongwei Chen, Jue Wang, Zheng Li, Christopher Wai Kei Lam, Ying Xiao, Qibiao Wu, Wei Zhang

**Affiliations:** 1 State Key Laboratory of Quality Research in Chinese Medicines, Macau University of Science and Technology, Avenida Wai Long, Taipa, Macau 999078, China; musthwchen@gmail.com (H.C.); wangjue2014must@gmail.com (J.W.); lizhengcpu@163.com (Z.L.); wklam@must.edu.mo (C.W.K.L.); 2 Faculty of Medicine, Macau University of Science and Technology, Avenida Wai Long, Taipa, Macau 999078, China; 3 Faculty of Chinese Medicine, Macau University of Science and Technology, Avenida Wai Long, Taipa, Macau 999078, China

**Keywords:** sugar-sweetened beverages, non-alcoholic fatty liver disease, systematic review, dose-response, meta-analysis

## Abstract

*Background:* Non-alcoholic fatty liver disease (NAFLD) is a serious health problem, but the dose-response relationship between sugar-sweetened beverages (SSBs) and NAFLD remains uncertain. *Methods:* A systematic review and dose-response meta-analysis were conducted following the PRISMA (Preferred Reporting Items for Systematic Reviews and Meta-Analyses) guidelines. Review Manager 5.3 and Stata 14.0 were used to combine trials and analyze data. The dose-response meta-analysis was performed by non-linear trend regression. *Results:* Twelve studies recruiting a total of 35,705 participants were included. The results showed that the consumption of SSBs was associated with 1.39-fold increased odds of NAFLD (95% CI: 1.29–1.50, *p* < 0.00001). The risk of NAFLD rose with an increased consumption of SSBs, while the consumptions of low doses (<1 cup/week), middle doses (1–6 cups/week) and high doses (≥7 cups/week) of SSBs increased the relative risk of NAFLD by 14%, 26% and 53%, respectively (*p* = 0.01, *p* < 0.00001, *p* = 0.03, respectively). *Conclusions:* This study demonstrates that consumers of SSBs are at significantly increased risk of NAFLD, and the consumption of SSBs has a dose-dependent effect on the risk of NAFLD. The findings of this study strengthen the evidence base for healthy dietary patterns and are meaningful for the primary prevention of NAFLD.

## 1. Introduction

Non-alcoholic fatty liver disease (NAFLD) is the most common chronic hepatic disease, characterized by steatosis and inflammation. NAFLD can progress to non-alcoholic steatohepatitis (NASH), which can lead to the development of liver cirrhosis or cancer in some patients [1,2,3]. The prevalence of NAFLD is estimated to be 20 to 30% in Western countries [4]. In China, NAFLD has also become a serious health problem with a community prevalence of about 15% [5].

Sugar-sweetened beverages (SSBs) are defined as beverages containing added caloric sweetener (high-fructose corn syrup, sucrose, etc.). SSBs, including soft drinks, fruit drinks, carbonated drinks and sport drinks, are one of the most common beverages second only to coffee and tea, and are the main source of artificially added sugar. It has been shown that the intake of SSBs is a risk factor for many medical conditions such as diabetes, obesity and metabolic syndrome [6,7,8,9,10], and may increase risk of mortality [11]. Moreover, SSBs have been reported to increase insulin resistance and inflammation, which play an important role in the development of NAFLD [12]. However, the results of different studies were inconsistent, with a few studies reporting that the patients with NAFLD had low consumption of SSBs [13], or that low doses of SSBs did not cause NAFLD [14,15]. Therefore, the conclusion still remains controversial. Furthermore, the dose-response relationship between the intake of SSBs and risk of NAFLD has never been systematically assessed. Therefore, it is necessary to further investigate the correlation of SSBs with NAFLD, and the dose-response relationship between them, aiming to provide more evidence for the prevention of NAFLD.

## 2. Methods

This systematic review and meta-analyses were performed following the PRISMA (Preferred Reporting Items for Systematic Reviews and Meta-Analyses) guidelines.

### 2.1. Search Strategy

A comprehensive literature search was carried out by two researchers (H.W. Chen and J. Wang). All English and Chinese language articles published to 20 January 2019 were screened from six databases including China National Knowledge Infrastructure (e conducted as follows (English database): (“Nonalcoholic Fatty Liver Disease”(Mesh Terms) OR (“NAFLD”(Title/Abstract) OR “Fatty Liver, Nonalcoholic”(Title/Abstract) OR “Nonalcoholic Steatohepatitis”(Title/Abstract) OR “Steatohepatitis, Nonalcoholic”(Title/Abstract) OR (“Nonalcoholic Fatty Liver”(Title/Abstract))) AND (“Carbonated Beverages”(Mesh Terms) OR (“Soft Drinks”(Title/Abstract) OR (“Carbonated drinks”(Title/Abstract) OR “Pop, Soda”(Title/Abstract)) OR “sugary soda”(Title/Abstract))). Chinese database (CNKI) searches: (“feijiujingxingzhifanggan” (non-alcoholic fatty liver disease)) AND ((“Tansuanyinliao” (carbonated drinks) OR “hantangqishui” (sugary soda) OR “hantangyinliao” (sugary beverages))).

The inclusion criteria were as follows: (1) observational studies (case-control, cross-sectional or cohort studies); (2) SSB consumption in relation to nonalcoholic fatty liver disease; (3) adjusted odds ratio (OR), risk ratio (RR), hazard ratios (HR) with coCNKI), PubMed, Web of Science, Medline, Cochrane library and EMBASE. The search details werrresponding 95% confidence intervals (CI) or other information sufficient for their calculation. We excluded the studies if their data or full texts were unavailable. Two researchers independently screened the titles and abstracts of articles. The full texts of all potentially relevant trials were retrieved for further assessment. Language was not limited. Two researchers independently evaluated the quality of each study using the Newcastle-Ottawa Quality Assessment Scale (NOS) [16]. Article quality was evaluated as 0–3 for low quality, 4–7 for moderate quality and ≥8 for high quality. Disagreements were resolved through discussion with a third reviewer.

### 2.2. Data Extraction

Data were extracted from identified articles by using a standardized extraction form. The important information was collected: (1) author names; (2) country; (3) year of publication; (4) sex of participants; (5) age of participants; (6) study design; (7) numbers of NAFLD cases, non-NAFLD cases and total number of participants; (8) SSB consumption category and SSB category; (9) confounder adjustment.

### 2.3. Statistical Analysis

Review Manager 5.3 (Nordic Cochrane Centre, Cochrane Collaboration, 2014 Copenhagen, Denmark) and Stata 14.0 (StataCorp., College Station, TX, USA) were used to combine trials and analyze data. We combined the RRs and their 95% CIs to conduct a new RR and 95% CI. Statistical heterogeneity was assessed by a Chi-squared test and *I*^2^ statistic [17]. Finally, a dose-response curve was derived by the collected relevant data.

In our study, all ORs and HR were roughly regarded as RR [18]. Twelve articles reported RR and adjusted effect estimates with 95% CIs. In those studies, “150–200 mL” was regarded as “a cup”, “1000 mL” as “6–7 cups”, “many” as “≥6–7 cups per week”. Intake doses were classified as low dose (<1 cup/week) meaning “seldom”, “rarely” or “less than monthly”; middle dose (1–6 cups/week) meaning “few”, “several times weekly”, “71 mL/day” or “3–4 cups/week”; and high dose (≥7 cups/week or 1 cup/day) meaning “≥1 cup per day”, “daily” or “more than once a day”. Subgroup analysis and sensitivity analysis were performed to evaluate the robustness of the results.

## 3. Results

**Figure 1:**
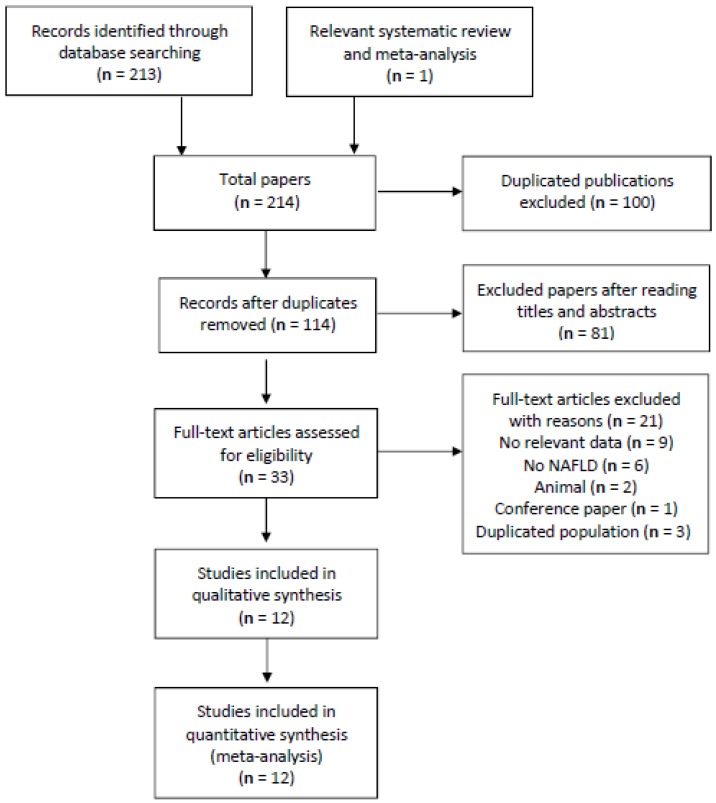
A summary of the studies selection process. NAFLD: Non-alcoholic fatty liver disease.

In total, 214 articles were identified through searching six different databases and relevant systematic reviews and meta-analyses. Subsequently, 114 articles were retrieved to Endnote X8 after duplicated articles were removed. The titles and abstracts were screened, and the full texts of potentially relevant 33 articles were retrieved for further assessment. Following to our inclusive and exclusive criteria, only 12 articles were included in our meta-analysis [13,14,15,19,20,21,22,23,24,25,26,27]. (Figure 1 describes the selection process.) The overall quality of included studies was moderate to high, 10 studies had 8 or more points (high-quality), and only two studies were of moderate quality (6 points) according to NOS standards. Most of the included studies in our meta-analysis adjusted the confounding factors, such as diabetes and body mass index (BMI) [13,14,21,22,24,26].

Characteristics of the included studies are shown in Table 1. These 12 studies included: a cohort study, two case-control studies and nine cross-sectional studies. Four studies were performed in China, and the others were conducted in USA, Germany, Australia and Israeli. All included studies recruited 35,705 participants. The consumption of SSBs ranged from <1 cup/week to ≥7 cups/week (the highest consumption was 4 cups/day).

SSB consumption in all studies was measured by the Harvard semi-quantitative food frequency questionnaire (FFQ), or physician-administered food frequency questionnaire.

### 3.1. The Risk of NAFLD in Patients Consuming Sugar-Sweetened Beverages

**Figure 2:**
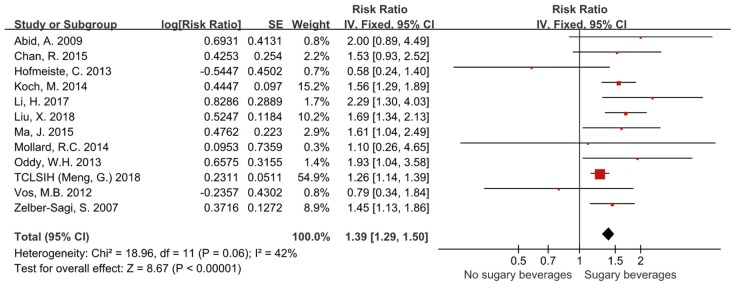
Forest plots showing that the consumptions of sugar-sweetened beverages increased the relative risk of NAFLD by 39%.

Twelve studies with 35,705 participants were included in the data analysis for risk of NAFLD. The pooled RR of NAFLD in individuals consuming sugar-sweetened beverages was 1.39 (95% CI, 1.29–1.50). The difference was statistically significant between groups (*p* < 0.00001) (Figure 2). There was no significant heterogeneity for this outcome (*I*² = 42%) and the fixed effects model was applied.

### 3.2. Subgroup Analyses on the Basis of Different Doses

**Figure 3:**
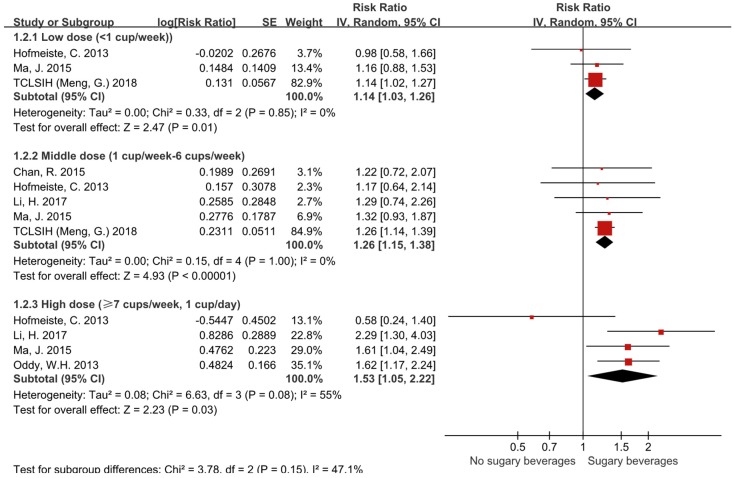
Forest plots showing that the consumptions of low doses (<1 cups/week), middle doses (1–6 cups/week) and high doses (≥7 cups/week) of sugar-sweetened beverages increased the relative risk of NAFLD by 14%, 26% and 53%, respectively.

As shown in Figure 3, the pooled RR of NAFLD in individuals consuming low doses of SSBs (<1 cup/week) was 1.14 (95% CI, 1.03–1.26). The RR of NAFLD in individuals consuming middle doses of SSBs (1–6 cups/week) was 1.26 (95% CI, 1.15–1.38). The RR of NAFLD in individuals consuming high doses of SSBs (≥7 cups/week) was 1.53 (95% CI, 1.05–2.22). The differences were statistically significant (*p* = 0.01, *p* < 0.00001, *p* = 0.03, respectively). Significant heterogeneity existed in high dose subgroup (*I*² = 55), and the random effects model was applied in the subgroup meta-analysis.

### 3.3. Assessment of Publication Bias

**Figure 4:**
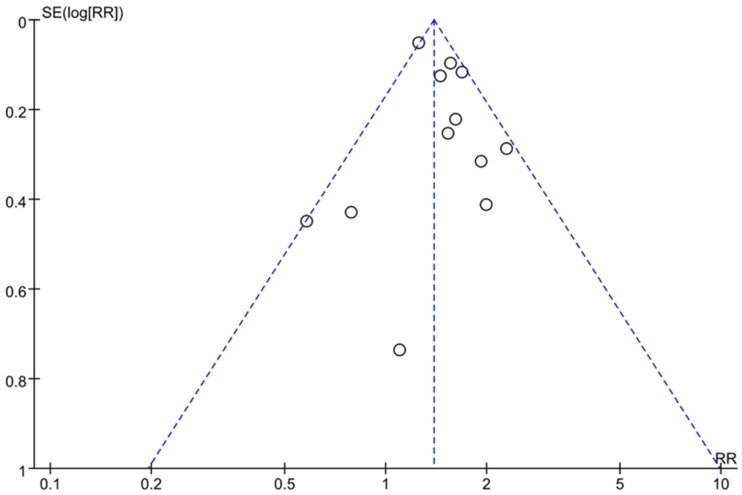
Funnel plots of all studies showing that publication bias existed.

As shown in Figure 4, the funnel plots were asymmetric. This result indicated that there might be publication bias in this study.

### 3.4. Sensitivity and Subgroup Analysis

In general, there was good homogeneity between all studies included in all meta-analyses. With regard to the correlation of SSB consumption with the risk of NAFLD, the primary endpoint, inclusion of all studies produced a statistically significant difference, showing that the consumption of SSBs was associated with a 1.39-fold increased odds of NAFLD (95% CI, 1.29–1.50, *p* < 0.00001, *I*² = 42). Sensitivity meta-analysis (after excluding two studies [13,14]) showed that the consumption of SSBs was associated with 1.59-fold increased odds of NAFLD (95% CI, 1.42–1.77, *p* < 0.00001, *I*² = 0). Subgroup analysis showed that low doses (RR = 1.14, 95% CI, 1.03–1.26), middle doses (RR = 1.26, 95% CI 1.15–1.38) and high doses (RR = 1.53, 95% CI, 1.05–2.22) of SSBs increased the relative risk of NAFLD by 14%, 26% and 53%, respectively (*p* < 0.05). There was no geographical heterogeneity between Asian and non-Asian populations, the consumption of SSBs increased the risk of NAFLD by 51% in Asian populations (95% CI: 1.27–1.79, *p* < 0.00001), and by 38% in non-Asian populations (95% CI: 1.05–1.83, *p* = 0.02). The results are shown in Table 2 and Supplementary Figure S1. A similar finding was noted when only 10 high-quality studies (NOS ≥ 8) were considered (RR = 1.38, 95% CI 1.28–1.49, *p* < 0.00001) (Table 2 and Supplementary Table S1), and when only six studies with at least 500 patients in each group were considered (RR = 1.44, 95% CI 1.17–1.77, *p* < 0.00001) (Table 2). These findings suggest that the results of overall analysis was robust for the correlation of SSB consumption with the risk of NAFLD.

### 3.5. Dose-Response Meta-Analysis

**Figure 5:**
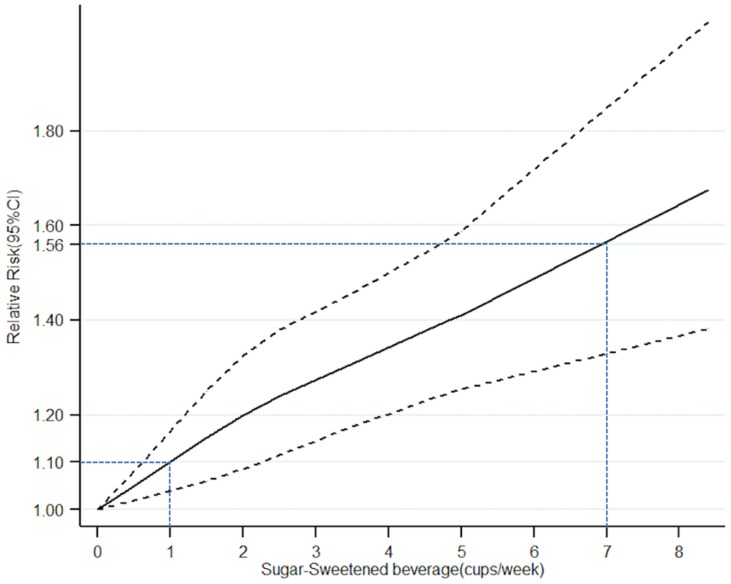
Association between frequency of sugar-sweetened beverages intake (cups/week) and risk of NAFLD obtained by dose–response meta-analyses. Solid line represents the estimated odds ratio and the dot-dashed lines represent the 95% confidence intervals.

The consumption of SSBs (cups/week) was expressed as an ordinal variable (Table 3). In addition, Tianjin Chronic Low-grade Systemic Inflammation and Health (TCLSIH) Cohort Study contained two studies [13,28]. The dose-response meta-analysis model revealed a non-linear relationship between SSBs (cups/week) and NAFLD (*p* for non-linear trend < 0.00001) (Figure 5). A monotonically increasing relationship was observed for the consumption of SSBs per week. (RR = 1.10 for 1 cup/week; RR = 1.56 for 7 cups/week (1 cup/day).

## 4. Discussion

This study is an updated systematic review and meta-analysis assessing the association between SSBs and risk of NAFLD. The results of this study demonstrated a statistically significant association between SSB consumption and the risk of NAFLD, which is consistent with the results of a previous study [29]. Our meta-analysis included 12 studies recruiting a total 35,705 participants, and summarized many more studies and larger sample sizes than the previous meta-analysis [29] (which included only four studies and 6326 participants), thus further confirming the results of the previous study and strengthening the evidence base for this topic.

The most important advantage of this meta-analysis is that, to the best of our knowledge, this is the first dose-response meta-analysis evaluating the dose-dependent effects of SSBs on risk of NAFLD. Our results showed that consumptions of low doses (<1 cup/week), middle doses (1–6 cups/week) and high doses (≥7 cups/week) of SSBs significantly increased the relative risk of NAFLD by 14%, 26% and 53%, respectively (*p* = 0.01, *p* < 0.00001, *p* = 0.03, respectively). Therefore, in order to prevent NAFLD, it is essential to stop the intake of sugary beverages, as even <1 cup/week of sugary beverages will significantly increase the risk of NAFLD by 14% (*p* = 0.01). Furthermore, the dose-response meta-analysis model revealed a non-linear relationship between SSBs (cups/week) and NAFLD (*p* value for non-linear trend is less than 0.00001). A monotonically increasing relationship was observed for the consumption of SSBs per week (RR = 1.10 for 1 cup/week; RR = 1.56 for 7 cups/week for 1 cup/day); However, this dose-response meta-analysis can merely reflect the trends of NAFLD in China, because all relevant data were obtained from Chinese population. [13,26,27,28].

Although the mechanisms of SSBs promoting NAFLD are still unclear, accumulated evidence has demonstrated that fructose might play a significant role [30]. Sugary beverages contain high fructose corn syrup, starch syrup, sucrose, and artificial sweeteners, and intake of fructose can increase the synthesis and deposition of liver triglyceride (TG) and reduce the clearance rate of TG [31]. A long-term high-fructose diet might lead to NAFLD [32]. Compared with the same calories of sucrose, the simultaneous intake of fructose and glucose causes more-serious fat depositions in the liver [33]. Meanwhile, the intake of fructose can significantly increase the expression of fructose kinase and fatty acid synthase in the livers of NAFLD patients [34], which accelerates fructose metabolism and fatty acid synthesis, and promotes lipid deposition in the liver, thus forming a vicious circle.

There are several epidemiological studies that have assessed the association between average fructose consumption and hepatic steatosis. Data from an observational study recruiting 2634 individuals showed that soft drink consumption was associated with an increased risk of fatty liver disease [15], especially in overweight and obese individuals [23]. Another study indicated that the consumption of fructose in NAFLD sufferers is about two- to three-fold higher than the matched controls [34]. A small cross-sectional study in Israel demonstrated that carbonated beverage consumption is a predictor of fatty liver [20]. In addition, another study found a link between high fructose consumption and visceral obesity in adolescents [35]. The most powerful evidence for fructose-induced fatty liver is a six-month randomized clinical trial which indicated that a relative change in liver fat content was significantly increased in the sucrose sweet drink group compared with non-calorie beverages [36].

The fructose in fruits is relatively safe and there is no need to deliberately reduce fruit consumption [37]. Small doses of fructose might be beneficial to health [38]. WHO-free sugars and their intake limit the American Heart Association recommended that the intake of sugar for adults be less than 5% of total energy (equivalent to 2.5% of energy from fructose), but the actual percentage is 14.6%, far exceeding the recommended limit [38].

However, our meta-analysis had some limitations. Most studies included in this meta-analysis were cross-sectional studies or case-control studies that did not incorporate the time sequence criteria for causality. The possible biases and confounders might not infer to causal inference [39]. The information of dietary habits was obtained through a food frequency questionnaire (FFQ) in some studies, which might lead to underreporting and interviewer bias. Furthermore, the information on SSB intake in some studies maybe over- or under-estimate the real daily consumption of sugary beverages [40]. Heterogeneity and potential risk of bias of the included studies might undermine the robustness of this study.

## 5. Conclusions

In summary, this updated systematic review and dose-response meta-analysis confirmed the correlation between SSB consumption and NAFLD, and further revealed that the consumption of SSBs has a dose-dependent effect on the risk of NAFLD. The findings of this study strengthen the evidence base for healthy dietary patterns and are meaningful for the primary prevention of NAFLD. Due to the intrinsic limitations of the included studies, more prospective randomized controlled trials (RCTs) are still needed to confirm the dose-response relationship of SSBs and NAFLD.

## Figures and Tables

**Table 1:** Characteristics of all studies included in this meta-analysis.

Author, Year	Country	Age/Sexes	Study Design	Cases/Non-Cases, Total Number	Sugar-Sweetened Beverage Consumption	Results, Adjusted OR (95% CI)	Exposure Definition and Exposure Measurement	Confounder Adjustment	Quality Score
Abid, A. 2009 [22]	Israel	43 ± 12 y, M/F	Cross-sectional study	60/30, 90	NA	2 (0.89, 4.47)	Soft drinks, FFQ	Adjusted for OR, age, sex, smoking habits, physical activity, dietary composition, BMI, metabolic syndrome, triglyceride, HOMA and metabolic biomarkers.	8
TCLSIH (Meng, G.) 2018 [13]	China	41.2 ± 11.9 y, 13,529 M/13,261 F	Cross-sectional study	NA, 26,790	0, <1 cup/week, ≥1 cup/week	1.26 (1.14, 1.4)	Soft drinks, FFQ	Adjusted for age, sex, BMI, OR, smoking status, drinker status, educational level, employment status, household income, family history of diseases, total energy intake, protein intake, carbohydrate intake, fat intake, EPA and DHA intake, physical activity and metabolic syndrome and consumption of other beverages.	8
Ma, J. 2015 [15]	USA	M ≥ 35 y, F ≥ 40y, M/F	Cross-sectional study	NA,2634	0, 1, 4, 10 servings/week	1.61 (1.04, 2.5)	SSBs, FFQ	Adjusted for age, sex, energy intake, alcohol intake, dietary fiber, dietary fat, dietary protein, SSBs or diet soda, smoking and Framingham cohort.	9
Li, H. 2017 [27]	China	45.15 ± 12.52 y, 325 M/80 F	Cross-sectional study	NA, 405	0, few (<1000 mL/week), many (>1000 mL/week)	2.29 (1.3, 4.03)	SSBs, FFQ	NA	6
Koch, M. 2014 [19]	Germany	67.6 y, M/F	Case-control study	171/183, 354	NA	1.56 (1.29, 1.88)	Soft drinks, FFQ	Adjusted for age and sex, smoking status, smoking duration, physical activity, total energy intake and years of education.	9
Vos, M.B. 2012 [25]	USA	12 ± 2.6 y, M/F	Cross-sectional study	NA, 149	>6 SSBs/week	0.79 (0.34, 1.87)	SSBs, FFQ	NA	6
Mollard, R.C. 2014 [21]	USA	15.4 ± 1.8 y, M/F	Cross-sectional study	21/47, 68	NA	1.1 (0.26, 4.67)	Soda, FFQ	Adjusted for available carbohydrate, fiber, protein, and total fat, carbohydrate, fiber, protein, total fat, age, and sex, BMI, ethnicity and cardiorespiratory fitness.	8
Chan, R. 2015 [26]	Hongkong, China	48.1 ± 10.6 y, 332 M/465 F	Case control study	220/577, 797	0, 1–71 mL/day, >71 mL/day	1.53 (0.93, 2.52)	SSBs, FFQ	Adjusted for age, gender, BMI, smoker status, drinker status, central obesity, triglyceride > 1.7 mmol/L, reduced HDL-cholesterol, hypertension, impaired fasting glucose or diabetes and PNPLA3 genotypes.	8
Zelber-Sagi, S. 2007 [20]	Israel	50.7 ± 10.4 y, 184 M/165 F	Cross-sectional study	108/241, 349	NA	1.4 (1.13, 1.85)	Soft drinks, FFQ	Adjusted for age, gender, BMI and total calories.	8
Oddy, W.H. 2013 [23]	Australia	14 y/17 y, M/F	Cohort study	151/844, 995	Fourth quartile of soft drinks	1.93 (1.04, 3.56)	Soft drinks, FFQ	Adjusted for western dietary pattern, healthy dietary pattern, sex, misreporting, TV viewing, frequency of physical activity and family income.	9
Liu, X. 2018 [24]	China	16–23 y, M/F	Cross-sectional study	221/1418,1639	NA	1.69 (1.34, 2.56)	Soft drinks, FFQ	Adjusted for age, sex, BMI, economic income, smoking status, educational level, physical activity, family history of diabetes and stroke and energy intake.	8
Hofmeiste, C. 2013 [14]	Germany	10–65 y, M/F	Cross-sectional study	374/1061, 1435	rarely/never, several times/months, several times/weeks, daily	0.58 (0.24, 1.40)	Soft drinks, FFQ	Adjusted for age, sex, BMI, WHR, sweets, metabolic syndrome, HTN and DM.	8

Abbreviations: BMI, body mass index; CI, confidence interval; DHA, Docosahexaenoic Acid; DM, diabetes mellitus; EPA, Eicosapentaenoic Acid; FFQ, food frequency questionnaire; HDL, high-density lipoprotein; HOMA, homeostasis model assessment; HTN, hypertension; M/F, male/female; NA, not applicable; NAFLD, non-alcoholic fatty liver disease; NASH, non-alcoholic steatohepatitis; OR, odds ratio; SSB, sugar-sweetened beverage; WHR, waist–hip ratio; y, year.

**Table 2:** Summary of subgroup meta-analyses.

Subgroups		Number of Studies	RR (95% CI)	Statistical Method	*p*-Value
All studies		12	1.39 (1.29–1.50)	fixed	<0.00001 *
Geographical location	Asian populations	6	1.51 (1.27–1.79)	random	<0.00001 *
	Non-Asian populations	6	1.38 (1.05–1.83)	random	0.02 *
Study design	Cross-sectional study	9	1.35 (1.24–1.46)	fixed	<0.00001 *
	Case-control study	2	1.56 (1.30–1.86)	fixed	<0.00001 *
	Cohort study	1	1.93 (1.04–3.58)	fixed	0.04 *
Sample size	≥500	6	1.44 (1.17–1.77)	random	0.0006 *
	<500	6	1.54 (1.34–1.77)	fixed	<0.00001 *
NOS ≥ 8	Yes	10	1.38 (1.28–1.49)	fixed	<0.00001 *
	No	2	1.41 (0.50–3.99)	random	0.52
Adjustment for Confounder	Yes	10	1.38 (1.28–1.49)	fixed	<0.00001 *
	No	2	1.41 (0.50–3.99)	random	0.52

Abbreviations: CI, confidence interval; NOS, Newcastle-Ottawa scale; RR, relative risk. *: statistically significant.

**Table 3:** Epidemiological studies of the intake frequency of sugar-sweetened beverages (cups/week) and risk of NAFLD.

Author	Intake Frequency (Cups/Week)	Midpoint Frequency (Cups/Week)	Case/*n*	OR (95% CI)
Li, H. 2017	0	0	80/199	1
	Few (<1000 mL/week) (<7 cups/week)	3.5	85/202	1.30 (0.74–2.26)
	Many (>1000 mL/week) (>7 cups/week)	8.4	127/281	2.29 (1.30–4.03)
TCLSIH (Meng, G.) 2017	Almost never	0	4076/14,985	1
	<1 cup/week	0.5	1211/4606	1.14 (1.02–1.27)
	≥1 cups/week	1.2	1971/7199	1.26 (1.14–1.40)
TCLSIH (Xia, Y.) 2019	0	0	466/1200	1
	2–3 cups/week	2.5	588/1200	0.93 (0.75–1.16)
	4–6 cups/week	5	614/1200	1.12 (0.90–1.39)
	≥1 cup/day (7 cups/week)	8.4	732/1200	1.40 (1.11–1.76)
Chan, R. 2015	0	0	87/231	1
	1–71 mL/day (0–4 cups/week)	2	47/163	1.22 (0.72–2.08)
	>71 mL/day (>4 cups/week)	4.8	86/183	1.53 (0.93–2.52)

Abbreviations: CI, confidence interval; OR, odds ratio. For the open-ended upper interval, we used 1.2-fold as its lower limit.

## Supplementary Materials

The following are available online at https://www.mdpi.com/1660-4601/16/12/2192/s1, Figure S1: Forest plots showing that the consumption of SSBs significantly increased the risk of NAFLD in both Asian (*p* < 0.00001) and non-Asian populations (*p* = 0.02), Table S1: Quality assessment of the included studies (Newcastle-Ottawa scale).
