# Supplementary material for: Consumption of Sugar-Sweetened Beverages Has a Dose-Dependent Effect on the Risk of Non-Alcoholic Fatty Liver Disease: An Updated Systematic Review and Dose-Response Meta-Analysis
Source: Int J Environ Res Public Health. 2019 Jun 21;16(12):2192. doi: 10.3390/ijerph16122192 (PMC6617076; doi:10.3390/ijerph16122192)
Supplement: Supplementary file 1 [file ijerph-16-02192-s001.zip › ijerph-498717-SI.pdf]

Supplementary Data

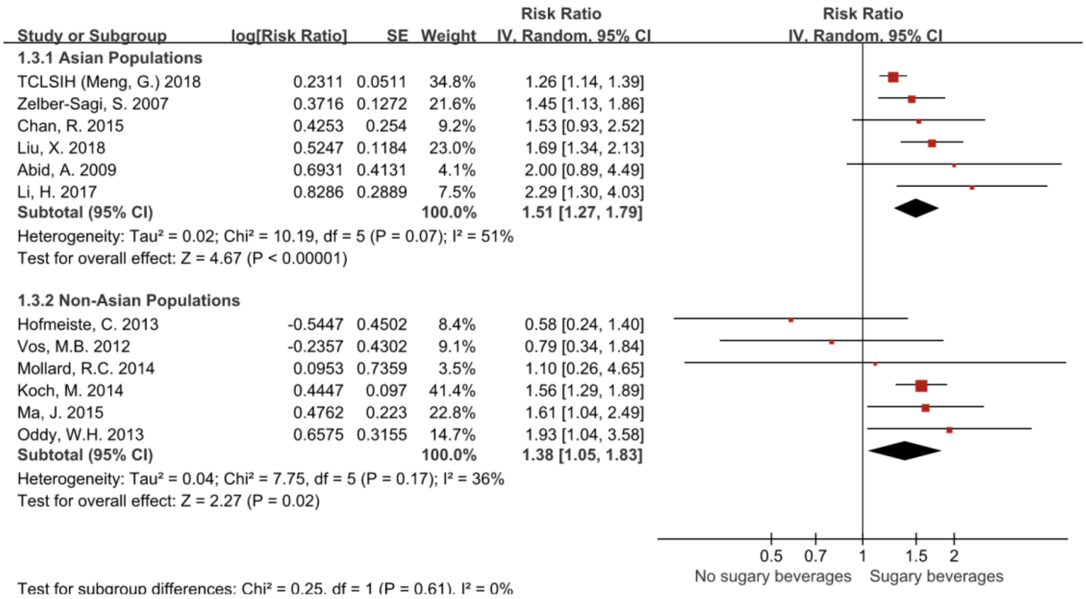

Supplementary Figure 1. Forest plots showing that the consumption of SSBs significantly increased the risk of NAFLD in both Asian ($P < 0.00001$) and non-Asian populations ($P = 0.02$).

Supplementary Table 1. Quality assessment of the included studies (Newcastle-Ottawa scale)

**Author, year**	**Quality assessment**			
	**Comparability**	**Selection**	**Outcome**	**Total score**
Abid, A. 2009	3	2	3	8
TCLSIH (Meng, G.) 2018	3	2	3	8
Ma, J. 2015	4	2	3	9
Li, H. 2017	3	0	3	6
Koch, M. 2014	4	2	3	9
Vos, M.B. 2012	3	0	3	6
Mollard, R.C. 2014	3	2	3	8
Chan, R. 2015	3	2	3	8
Zelber-Sagi, S. 2007	3	2	3	8
Oddy, W.H. 2013	4	2	3	9
Liu, X. 2018	3	2	3	8
Hofmeiste, C. 2013	3	2	3	8
